# Functional Movement Disorder in Familial Ataxia: A Case Report of Monozygotic Twins

**DOI:** 10.1002/mdc3.70196

**Published:** 2025-06-23

**Authors:** Daniela Kern, Christian Windpassinger, Petra Schwingenschuh

**Affiliations:** ^1^ Department of Neurology Medical University of Graz Graz Austria; ^2^ Institute of Human Genetics Medical University of Graz Graz Austria

**Keywords:** functional movement disorder, functional neurological disorder, tremor, ataxia, CACNA1C gene

Functional neurological disorder (FND) is commonly seen as a comorbidity of movement disorders.^1^ We here present the case of monozygotic twins (A and B) with familial ataxia, one of whom (A) experienced FND‐comorbidity.

## Case Report

A 43‐year‐old white European male (twin A) presented with tremor and gait disturbance. Five years ago, 1 week after undergoing cardiac ablation for tachycardia, he experienced a panic attack. The following day, he developed an abrupt gait disturbance. Although his presentation was consistent with a FND‐pattern, specifically, an ataxic gait that improved with distraction, this diagnosis was not established at the time. Due to progressive deterioration, he was admitted to an inpatient‐rehabilitation‐facility 3 years ago, marking his first time away from home. He reported anxiety and pronounced fatigue, which was overlooked throughout the intensive rehabilitation‐program. During a demanding physiotherapy session, he experienced an acute onset of a postural‐kinetic tremor of both upper limbs. The tremor was attenuated by cognitive distraction and disappeared during motor tasks. It became chronic with diurnal fluctuations. Besides gait disturbance and tremor, neurological examination revealed dysarthria and impaired initiation of voluntary saccades, accompanied by excessive blinking (see Video 1). These findings were interpreted as oculomotor apraxia. However, similar oculomotor findings have also been reported in patients with FND^2^.

**Video 1:** Segment 1: Excessive blinking during the examination of the saccades, this could indicate oculomotor apraxia but it is also seen in patients with FND. Segment 2: Dysarthria. Segment 3: Functional tremor with cognitive (counting backwards) and motor distractibility. Segment 4: Functional gait disorder with scissoring gait.

Twin B presented with an antalgic gait attributable to lumbar disc herniation; however, no features suggestive of a functional gait disorder were observed. Furthermore, there was no evidence of functional tremor. Neurological examination revealed dysarthria, impaired initiation of voluntary saccades, and mild intention tremor (see Video 2).

**Video 2:** Segment 1: Dysarthria. Segment 2: Impaired initiation of voluntary saccades. Segment 3: Mild limb ataxia and intention tremor. Segment 4: Antalgic gait pattern due to lumbar disc herniation. Segment 5: Impaired tandem gait.

Medical history revealed learning disability, apical hypertrophic cardiomyopathy (HCM), and chronic urinary retention in the twins (A and B) and in a milder form in their brother and sister. The patient's mother had died of sudden cardiac death at a young age. The father and two sisters were asymptomatic.

A 3‐Tesla‐MRI of the brain and cervical spine and nerve‐conduction‐studies were normal. Genetic testing for Friedreich ataxia was negative. In addition to the cardiac work‐up, a genetic analysis for known HCM‐associated genes was performed, revealing a heterozygous missense variant in the CACNA1C‐gene (c.4132G>A,p.(Gly1378Arg)). This rare variant is located in an evolutionary conserved region of the gene and is not listed in population databases such as GnomAD.^3^ Additionally, the variant is predicted to be deleterious by numerous prediction tools. Segregation analysis revealed that the variant is also present in the affected siblings, but not in one of the asymptomatic sisters. The other asymptomatic sister and the father refused genetic testing.

Tremor and gait disorder showed positive signs of FND. First, both symptoms had an acute onset after a precipitating event. Furthermore, the tremor was distractible, showed entrainment and variability.^4^ The gait pattern was inconsistent when examined and showed patterns of functional gait disorder.^5^

We diagnosed FND‐comorbidity in twin A on top of a familial ataxic syndrome and explained the diagnosis to the patient and his family. We recommended specialized physical and occupational therapy.

## Discussion

We present the case of monozygotic twins with a progressive, familial multisystemic syndrome, in which only twin A had an acute exacerbation of the movement disorder and was diagnosed with FND‐comorbidity.

The twins share the same biological predisposing factors for FND, in particular presence of movement disorder^6^ and learning disability.^7^ Both patients were suspected to exhibit autistic traits, although no formal diagnosis was made. Notably, autistic traits are highly prevalent in patients with FND, with emerging evidence suggesting shared pathophysiological mechanisms between both disorders.^8^

Precipitating factors varied between the twins. It was only twin A, who underwent cardiac ablation, which was followed by a panic attack. The majority of patients with FND report physical events that precede FND, often accompanied by panic symptoms.^9^ The tremor emerged during a rehabilitation‐program that he experienced as highly stressful, due to factors like being away from home, physical overexertion and anxiety. In contrast, twin B did not report similar stressful life events, major medical procedures or panic attacks. He experienced pain because of lumbar disc herniation, but this did not cause neurological deficits or require surgery. In conclusion, despite being monozygotic twins, the two patients exhibited significantly different biopsychosocial risk profiles. (See Table 1 for a detailed presentation of biological, psychological and socioeconomic risk factors).

**TABLE 1:** Biological, psychological and socioeconomic risk factors in both twins

Risk factors	Age/Status	Twin A	Twin B
Biological risk factors/medical history	Pregnancy and Birth	Unremarkable	Unremarkable
	School Age	Learning disability Balance disturbance	Learning disability Balance disturbance
	35–36 years	Chronic urinary retention—intermittent self‐catheterization	Chronic urinary retention—intermittent self‐catheterization
	37 years	Hypertrophic cardiomyopathy—conservative treatment	Hypertrophic cardiomyopathy—conservative treatment
	38 years	Cardiac ablation for supraventricular tachycardia, followed by panic attack and functional gait disorder	‐
	40 years	Inpatient rehabilitation—sudden onset of functional tremor	‐
	43 years	‐	Lumbar disc herniation—conservative treatment
Psychological risk factors	Childhood	No history of trauma or abuse	No history of trauma or abuse
	School Age	Suspected autistic traits, no formal diagnosis	Suspected autistic traits, no formal diagnosis
	38 years	Panic attack, followed by anxiety symptoms	–
Socioeconomic risk factors	Education:	Special needs school (6 to 15 years)	Special needs school (6 to 15 years)
	Profession:	Locksmith's Assistant: early retirement at the age of 38 due to cardiac symptoms.	Gardener's Assistant: still working in this profession.
	Living Situation:	Twin A and B share a small apartment in a rural area and receive support from one of their sisters.	Twin A and B share a small apartment in a rural area and receive support from one of their sisters.
	Relationship Status:	Has never been in a relationship.	Has never been in a relationship.
	Socioeconomic Status:	Raised in a family of six children, grew up in modest circumstances. He now lives on disability benefits.	Raised in a family of six children, grew up in modest circumstances. He now earns his own income.

The cause of the multisystemic syndrome has yet to be elucidated. To date, we do not have sufficient data to accurately determine the causal potential of the CACNA1C‐gene variant. However, other mutations in the CACNA1C‐gene have also been associated with neurological phenotypes, including developmental delay, intellectual disability, autism, and motor disorders.^10^

This case highlights the multifactorial etiology of FND within a biopsychosocial framework. Even monozygotic twins sharing the same biological preposition may differ in their psychosocial risk factors, resulting in only one of them developing FND. In our case, we had the advantage of comparing the symptoms of two monozygotic twins, with an assumed identical time course of a genetic syndrome. Nevertheless, in case of acute worsening of a chronic, progressive movement disorder, it is important to examine for FND‐comorbidity.

## Author Roles

(1) Research project: A. Conception, B. Organization, C. Execution; (2) Data Collection: A. Clinical Assessment, B. Genetic Analysis, C. Literature Review; (3) Manuscript Preparation: A. Writing of the first draft, B. Review and Critique.

D.K.: 1B, 1C, 2A, 2C, 3A.

C.W.: 1B, 1C, 2B, 3B.

P.S.: 1A, 1B, 1C, 2A, 2C, 3B.

## Disclosures

**Ethical Compliance Statement:** No formal approval by an ethics committee was required. The Journal's Ethical Publication guidelines and the Declaration of Helsinki were followed. Informed written consent on video acquisition and case publication was obtained from the patient using our standardized patient's video consent form. We confirm that we have read the Journal's position on issues involved in ethical publication and affirm that this work is consistent with those guidelines.

**Funding Sources and Conflict of Interest:** No specific funding was received for this work. The authors declare that there are no conflicts of interest relevant to this work.

**Financial Disclosures for the previous 12 months:** The authors declare that there are no additional disclosures to report.

## Data Availability

The data that support the findings of this study are available from the corresponding author upon reasonable request.
